# Cardiac stem cells and their clinical use

**DOI:** 10.1590/S1679-45082014MD2739

**Published:** 2014

**Authors:** Jacyr Pasternak

**Affiliations:** 1 Hospital Israelita Albert Einstein, São Paulo, SP, Brazil

**Keywords:** Stem cell transplantation, Coronary disease/therapy, Myocarditis/therapy, Treatment outcome

## Abstract

Stem cells have been used to correct the ravages of atherosclerotic heart disease and other diseases that involve acute deficit of myocardial muscle, such as myocarditis. Previous attempts at using bone marrow derived from stem cells have not been particularly successful. New technologies using cardiac stem cells seem to offer a better perspective of obtaining such regeneration.

## RESUMO

Células-tronco têm sido usadas para regeneração miocárdica, em casos de complicações da aterosclerose e em doenças como miocardites, nas quais há profunda perda da musculatura miocárdica. Tentativas de provocar essa regeneração com células-tronco originadas da medula óssea não foram particularmente bem-sucedidas. Novas tecnologias usando células-tronco de origem miocárdica parecem oferecer uma melhor perspectiva de obter tal regeneração.

The use of stem cells in an attempt to regenerate an injured heart has been done for a long time; the first research on this topic dates back to 2003^(1)^ describe cells collected from bone marrow. The abilities of bone marrow stem cells are well known, which after a while are identified to be present in circulating blood, especially during complete regeneration of all hematopoietic tissue and the immune system.

Although many studies have adopted this methodology, some studies have obtained less favorable results,^(2,3)^ but other studies have yielded more positive findings.^(4,5)^ There are several reasons for the differences among these results: different methodologies of preparing bone marrow-derived cells; doubts on what type of cell – mesenchymals or stem cell – is really relevant; different methods of infusions for these cells (intracoronary and intracardiac); different times between cardiac infarction as a factor to indicate these treatments and their accomplishments; and different methodologies to measure results.^(6)^

A 2010 review of clinical studies showed some problems, particularly related to clinical results analysis. In general, almost all of these studies in this review included few patients and used several methods, the most objective method being measurement of left ventricular ejection fraction with temporal ranges postinfarction. The clinical impressions are subjected to the famous Hawthorne effect (if the medical team and the patient expect an improvement, they will see such improvement…).^(7)^

The use of allogeneic mesenchymal cells, which do not induce graft-*versus*-host disease, showed a relatively small, but realistic, improvement post-infarction compared with autologous mesenchymal cells.^(8)^

Other types of stem cells have also been used, such as cells obtained from fat tissue, in experimental models.^(9)^ A randomized study with more patients, also relatively new, did not show enthusiastic findings in the use of bone marrow-derived cells to improve clinical results postinfarction or in severe cardiac insufficiency.^(10)^

More recent studies have shown that cardiac stem cells have similar features as bone marrow cells. These cells are clonogenic and have the ability to differentiate themselves from cardiomyocytes, vascular smooth muscle cells, and endothelial cells. Animal model studies have shown better success with the use of such cells in regeneration of cardiac tissue after infarction. In addition, a recent study^(11)^ has shown encouraging findings in the use of cardiac stem cells, although they performed the study on a small group of patients. In that study, the Stem Cell Infusion in Patients with Ischemic Myocardiopathy (SCIPIO), cardiac stem cells were infused in 16 patients; 7 of them were designated as the control group. SCIPIO was not a closed study; instead, it was a randomized “open” study with all criticisms that this type of model could have. Cardiac stem cells, which have a cell surface marker called the c-kit, were obtained through auricular biopsies during cardiac surgery: tissues were expanded *in vitro* and were divided with a cell sorter and an immunomagnetic selection with micro pearls. Two million cells were collected from each patient and then were infused four months later. Improvements were seen in functional tests, and the extension of infarction was measured with magnetic resonance imaging, which was significant. The SCIPIO study is ongoing. If analysis of more cases can confirm these results, then the possibility exists that ischemic heart injuries can be repaired and, perhaps – it should be further investigated – heart disease from other causes.

Different from stem cells, cardiac stem cells apparently can expand without loss of its “stem effect” (*i.e.*, they retain themselves as stem cells). Such a phenomenon does not occur with bone marrow cells, which could be expanded but gradually become non-stem cells.

There are better proposals (but they are still proposals) that were never performed in humans. An example is the use of adult stem cells transformed into embryonic cells. Published experimental research and even clinical investigations using this technique are unknown.

Ischemic heart disease is the most common cause of death in developed countries and in countries such as Brazil, which rapidly reach the consumption standard of developed nations, including changes for the worse in diet. Although prevention is most effective for ischemic heart disease *versus* treatment after its occurrence, belief exists that treatment will be needed for complications resulting from this disease.

Results of new studies render hope that cardiac regeneration is clinically possible.
